# Metabolic Flux Analysis—Linking Isotope Labeling and Metabolic Fluxes

**DOI:** 10.3390/metabo10110447

**Published:** 2020-11-06

**Authors:** Yujue Wang, Fredric E. Wondisford, Chi Song, Teng Zhang, Xiaoyang Su

**Affiliations:** 1 Department of Medicine, Rutgers-Robert Wood Johnson Medical School, New Brunswick, NJ 08901, USA; yw429@rwjms.rutgers.edu (Y.W.); few11@rwjms.rutgers.edu (F.E.W.); 2 Metabolomics Shared Resource, Rutgers Cancer Institute of New Jersey, New Brunswick, NJ 08903, USA; 3 Division of Biostatistics, College of Public Health, The Ohio State University, Columbus, OH 43210, USA; song.1188@osu.edu; 4 Department of Mathematics, University of Central Florida, Orlando, FL 32816, USA; Teng.Zhang@ucf.edu

**Keywords:** metabolic flux analysis, MFA assumptions, tracer selection, non-steady-state versus steady-state

## Abstract

Metabolic flux analysis (MFA) is an increasingly important tool to study metabolism quantitatively. Unlike the concentrations of metabolites, the fluxes, which are the rates at which intracellular metabolites interconvert, are not directly measurable. MFA uses stable isotope labeled tracers to reveal information related to the fluxes. The conceptual idea of MFA is that in tracer experiments the isotope labeling patterns of intracellular metabolites are determined by the fluxes, therefore by measuring the labeling patterns we can infer the fluxes in the network. In this review, we will discuss the basic concept of MFA using a simplified upper glycolysis network as an example. We will show how the fluxes are reflected in the isotope labeling patterns. The central idea we wish to deliver is that under metabolic and isotopic steady-state the labeling pattern of a metabolite is the flux-weighted average of the substrates’ labeling patterns. As a result, MFA can tell the relative contributions of converging metabolic pathways only when these pathways make substrates in different labeling patterns for the shared product. This is the fundamental principle guiding the design of isotope labeling experiment for MFA including tracer selection. In addition, we will also discuss the basic biochemical assumptions of MFA, and we will show the flux-solving procedure and result evaluation. Finally, we will highlight the link between isotopically stationary and nonstationary flux analysis.

## 1. Introduction

Metabolism involves a set of chemical reactions that convert nutrients to energy and macromolecules to sustain cellular survival and growth. Many enzymes are thought to work on production lines: the product coming from the first enzyme is the substrate for the second enzyme. Together, these enzymes form metabolic pathways that fulfill certain metabolic tasks. The catabolic pathways, for example, break down large molecules such as proteins and lipids to make ATP and other small molecule metabolites. The anabolic pathways use biochemical energy stored in ATP to synthesize macromolecules. Interestingly, the catabolism and anabolism are overlapped rather than exclusive. Some enzymes, such as the fructose-bisphosphate aldolase, are shared between the catabolic glycolysis pathway and the anabolic gluconeogenesis pathway. Meanwhile, co-factors such as ATP, acetyl-CoA and NAD(PH) are shared among many enzymatic reactions [1]. These features demonstrate the fact that these biochemical reactions are highly interconnected and essentially form a network. The elucidation of the metabolic network was a huge scientific achievement in the 20th century [2]. Despite the vast knowledge on the structure of the metabolic network, the dynamic partition of metabolic material through different pathways under various physiological and pathological conditions remains incompletely characterized.

In the past two decades, metabolic research has been rapidly expanded due to the huge improvement on technologies for metabolite measurements. These technological advances include nuclear magnetic resonance (NMR) and mass spectrometry (MS) [3,4,5,6,7,8,9]. The large-scale analysis of the metabolite pool sizes, namely metabolomics, has been widely deployed in clinical research and pharmacology [10]. Metabolites can serve as biomarkers for diagnosis. For example, elevated spermine level and decreased citrate level in the prostate fluid has been shown to be a distinct metabolomics profile in prostate cancer patients [11,12]. Although these discoveries are highly valuable for diagnosis, the measurement of metabolite pool size alone reveals little information regarding the biochemical events behind such changes [13,14]. The increased pool size of a metabolite can be the result of either enhanced production or diminished consumption. For example, sildenafil is an inhibitor of phosphodiesterase type 5, which breaks down cyclic guanosine monophosphate (cGMP). Usage of sildenafil increases the pool size of cGMP but the flux through this metabolite is decreased [15]. This example demonstrates that changes in metabolite pool size do not directly translate into flux changes. An analogy for the relationship between the metabolite pool size and metabolic flux is the car traffic. The metabolite pool size represents the density of cars on the road while the flux represents the flow of traffic. Although the density of cars can be measured by taking a static picture of the road, such a measurement alone is insufficient to tell how fast the flow of traffic is.

Unlike the pool size of metabolites that can be directly measured by NMR and MS, the metabolic fluxes are usually unmeasurable. Although the extracellular fluxes such as nutrient uptake rates can be calculated based on the concentration changes over time, the intracellular fluxes cannot be measured this way. Instead, they have to be solved by the approach named flux analysis [16,17,18,19]. Flux analysis is a powerful technique and has been applied in multiple fields of research such as pharmaceutical production, drug design and cancer biology [20,21,22]. For example, about one century ago, Warburg et al. [23] discovered cancer cells generate more lactate than healthy cells under aerobic conditions, a phenomenon now well known as the Warburg effect [24]. However, it remains unclear for many decades how cancer cells could make use of lactate to support its own growth. Recently, with the help of metabolic flux analysis, it became clear that lactate is an important carbon source for citric acid cycle in both healthy and cancer cells [25,26].

Depending on what experimental data are available, the flux analysis can be carried out in different ways. However, all flux analysis methods must resolve one basic problem: the total number of unknown fluxes often exceed the total number of measurement (i.e., the network is undetermined). One way to resolve this issue is by simplifying the flux network and using stoichiometry balance of the intracellular metabolites to reduce the total number of unknown fluxes. This approach is known as stoichiometric flux analysis (SFA) [16,27,28,29]. On the other hand, if a detailed flux network has to be used, one could also resolve this issue by assuming a cellular objective function (e.g., maximum cell growth or ATP production). This approach is known as the flux balance analysis (FBA) [17,30,31,32,33,34]. In addition to satisfying the stoichiometric constraints, the intracellular fluxes must also maximize the objective function. 

Despite the popular application of FBA and SFA in system biology and metabolic engineering, these methods have some apparent limitations: (1) it is hard to estimate the fluxes through parallel pathways, cyclic pathways or bidirectional reversible reactions [17,35,36]. (2) The objective function for mammalian cell metabolism is hard to define. Mammalian cells, unlike bacteria in a batch culture, can exhibit different metabolic phenotypes during proliferation, differentiation and senescence [37]. These practical limitations largely come from the limited amount of measurements that are utilized in FBA and SFA. In contrast, metabolic flux analysis (MFA) uses stable isotope tracers and takes labeling patterns of intracellular metabolites as additional input information. While more commonly referred to as ^13^C-MFA, the framework of MFA is not limited to ^13^C labeling. In fact, MFA has been applied to analyze labeling from ^15^N, ^2^H and other tracers [14,38]. As a result, MFA is much more powerful than FBA and SFA in overcoming the two limitations mentioned above [17]. Table 1 has listed some most commonly used software tools for MFA. In this review, we will demonstrate how MFA is carried out, and we will discuss the principles of using MFA to solve metabolic fluxes.

## 2. Example on Upper Glycolysis

**Figure 1:**
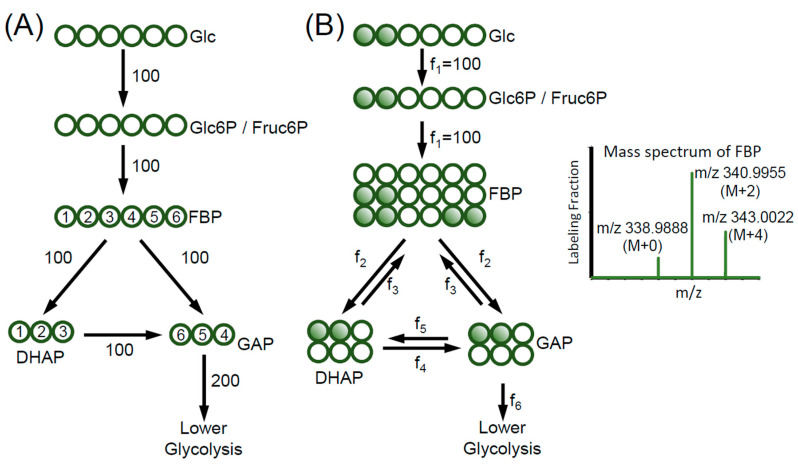
Comparison of stoichiometric flux analysis (SFA) and metabolic flux analysis (MFA). (**A**) For SFA, no isotope tracer is used. In the upper glycolysis network, the consumption rate of glucose and the production rate of GAP is 100 nmol/h and 200 nmol/h, respectively. The atoms of FBP, DHAP and GAP are numbered to illustrate the atom transition. (**B**) For MFA, 1,2-^13^C_2_-glucose is used as the tracer. As the result, FBP is labeled as M+0, M+2 and M+4. This labeling pattern indicates that the aldolase and triose phosphate isomerase have reverse fluxes, which are f_3_ and f_5,_ respectively. The abbreviations used in the figure are: Glc, glucose; Glc6P, glucose-6-phosphate; Fruc6P, fructose-6-phosphate; FBP, fructose 1,6-bisphosphate; DHAP, dihydroxyacetone phosphate and GAP, glyceraldehyde-3-phosphate. The fluxes are in the unit of nmol/h.

Without using isotope tracers, we have a very limited insight on how metabolites interconvert in the metabolic network. Taking upper glycolysis pathway as an example. If glucose is consumed at the rate of 100 nmol/h, glyceraldehyde-3-phosphate (GAP) goes to lower glycolysis at the rate of 200 nmol/h (Figure 1A). No more information on the metabolic fluxes can be drawn from this unlabeled experiment. In comparison, when 1,2-^13^C_2_-Glucose (Glucose where C-1 and C-2 are labeled with ^13^C) tracer is used (Figure 1B), we can observe the reversibility of upper glycolysis reactions. The fructose 1,6-bisphosphate (FBP) will be labeled in three forms, M+0 (no ^13^C labeling), M+2 (2 carbon atoms are labeled with ^13^C) and M+4 (4 carbon atoms are labeled with ^13^C) (Figure 1B). FBP can be made from the glucose tracer through the intermediates glucose-6-phopshate (Glc6P) and fructose-6-phopshate (Fruc6P). Both intermediates would have the same labeling (M+2) as the glucose tracer since their production only involves phosphorylation and isomerization without breaking the C-C bonds. Therefore, the M+2 FBP represents its synthesis flux mostly directly from Fruc6P (f_1_; Figure 1B). The observation of M+0 and M+4 FBP, however, suggests the presence of additional synthesis route for FBP, which involves recombination of the carbon backbones. Indeed, the aldolase reaction that breaks down FBP into dihydroxyacetone phosphate (DHAP) and GAP is reversible (f_2_ and f_3_; Figure 1B). The observed M+4 FBP has to be generated from M+2 DHAP and M+2 GAP through the reverse reaction of aldolase (f_3_; Figure 1B). Similarly, M+0 FBP has to be generated from M+0 DHAP and M+0 GAP. However, since the DHAP derived from Glucose C1–C3 (Glc[1-3]) must be M+2 labeled, there has to be an alternative way to generate M+0 DHAP. In fact, the triose phosphate isomerase (TPI) reaction is also reversible (f_4_ and f_5_; Figure 1B) converting M+0 DHAP from M+0 GAP. Thus, using isotopic tracers, we have established a qualitative link between the labeling pattern of FBP and the reversibility of the aldolase and TPI reactions. The lower the observed FBP M+2, the higher the reverse flux of aldolase (f_3_) is; the higher the observed FBP M+0, the higher the reverse flux of TPI (f_5_) is. Such reversibility of aldolase and TPI would not have been measurable without using an isotope labeled glucose tracer.

Although the real metabolic network might be more complicated than we illustrated (e.g., the existence of pentose phosphate pathway), this simple example illustrates the gist of MFA. When we use the suitable isotope labeled tracer, the labeling patterns of intercellular metabolites reflect the fluxes. The goal of MFA is to infer the metabolic fluxes from these isotope labeling patterns. Before we move to the solving process of MFA, we need to examine quantitatively how the labeling patterns are determined by the fluxes and what information is encoded in the labeling patterns.

When the cells are at metabolic steady state, the production rate of an intercellular metabolite equals the consumption rates of it. Furthermore, when the cells are under isotopic steady state, the production rate of a specific labeling fraction equals the consumption rates of it. For FBP, such mass balances on the labeled fractions are described by the following equation.

(1) $$\begin{array}{c} \{\begin{array}{c} f_{3}\times {\left(\mathrm{DHAP}⊕\mathrm{GAP}\right)}_{M+0}+f_{1}\times Glc_{M+0}=f_{2}\times FBP_{M+0}\\ f_{3}\times {\left(\mathrm{DHAP}⊕\mathrm{GAP}\right)}_{M+2}+f_{1}\times Glc_{M+2}=f_{2}\times FBP_{M+2}\\ f_{3}\times {\left(\mathrm{DHAP}⊕\mathrm{GAP}\right)}_{M+4}+f_{1}\times Glc_{M+4}=f_{2}\times FBP_{M+4} \end{array} \end{array}$$

In Equation (1), we use DHAP⊕GAP to represent the combination of GAP and DHAP. In such a condensation reaction, the substrates are combined independently in terms of their labeling patterns. Therefore, we have the following equations.

(2) $$\begin{array}{c} {\left(\mathrm{DHAP}⊕\mathrm{GAP}\right)}_{M+0}=DHAP_{M+0}\times GAP_{M+0}\\ {\left(\mathrm{DHAP}⊕\mathrm{GAP}\right)}_{M+2}=DHAP_{M+2}\times GAP_{M+0}+DHAP_{M+0}\times GAP_{M+2}\\ {\left(\mathrm{DHAP}⊕\mathrm{GAP}\right)}_{M+4}=DHAP_{M+2}\times GAP_{M+2} \end{array}$$

Our first observation is that Equation (1) is a set of linear equations. We can re-write them as

(3) $$\begin{array}{c} f_{3}\times \left[\begin{array}{c} {\left(\mathrm{DHAP}⊕\mathrm{GAP}\right)}_{M+0}\\ {\left(\mathrm{DHAP}⊕\mathrm{GAP}\right)}_{M+2}\\ {\left(\mathrm{DHAP}⊕\mathrm{GAP}\right)}_{M+4} \end{array}\right]+f_{1}\times \left[\begin{array}{c} {\mathrm{Glc}}_{M+0}\\ {\mathrm{Glc}}_{M+2}\\ {\mathrm{Glc}}_{M+4} \end{array}\right]=f_{2}\times \left[\begin{array}{c} {\mathrm{FBP}}_{M+0}\\ {\mathrm{FBP}}_{M+2}\\ {\mathrm{FBP}}_{M+4} \end{array}\right] \end{array}$$

The second observation is, due to the mass balance of FBP, the sum of the production fluxes equals the consumption flux

(4) $$\begin{array}{c} f_{1}+f_{3}=f_{2} \end{array}$$

Therefore, we can replace the consumption flux of FBP with the sum of the production fluxes, and Equation (3) can be written as

(5) $$\begin{array}{c} f_{3}\times \left[\begin{array}{c} {\left(\mathrm{DHAP}⊕\mathrm{GAP}\right)}_{M+0}\\ {\left(\mathrm{DHAP}⊕\mathrm{GAP}\right)}_{M+2}\\ {\left(\mathrm{DHAP}⊕\mathrm{GAP}\right)}_{M+4} \end{array}\right]+f_{1}\times \left[\begin{array}{c} {\mathrm{Glc}}_{M+0}\\ {\mathrm{Glc}}_{M+2}\\ {\mathrm{Glc}}_{M+4} \end{array}\right]=\left(f_{3}+f_{1}\right)\times \left[\begin{array}{c} {\mathrm{FBP}}_{M+0}\\ {\mathrm{FBP}}_{M+2}\\ {\mathrm{FBP}}_{M+4} \end{array}\right] \end{array}$$

Equation (5) shows that the labeling of FBP is the flux-weighted average of its substrates’ labeling (i.e., the higher the ratio of f_1_/f_3_, the similar the labeling pattern of FBP to that of Glc). If we move the FBP labeling vector to the left side of the equation, we have:

(6) $$\begin{array}{c} \left[\begin{array}{c} \begin{array}{cc} \begin{array}{c} {\mathrm{Glc}}_{M+0}-{\mathrm{FBP}}_{M+0}\\ {\mathrm{Glc}}_{M+2}-{\mathrm{FBP}}_{M+2}\\ {\mathrm{Glc}}_{M+4}-{\mathrm{FBP}}_{M+4} \end{array} & \begin{array}{c} {\left(\mathrm{DHAP}⊕\mathrm{GAP}\right)}_{M+0}-{\mathrm{FBP}}_{M+0}\\ {\left(\mathrm{DHAP}⊕\mathrm{GAP}\right)}_{M+2}-{\mathrm{FBP}}_{M+2}\\ {\left(\mathrm{DHAP}⊕\mathrm{GAP}\right)}_{M+4}-{\mathrm{FBP}}_{M+4} \end{array} \end{array} \end{array}\right]\left[\begin{array}{c} f_{1}\\ f_{3} \end{array}\right]=0 \end{array}$$

If we know DHAP⊕GAP, Glc and FBP, we can plug these values into Equation (6) and solve for the ratio between f_1_ and f_3_. In fact, all three equations of M+0, M+2 and M+4 fractions will give the same f_1_/f_3_ value. It should be noted that it is not the absolute value of f_1_ and f_3_ but the ratio of them affects the equation. In fact, f_1_ can take any value to fulfill Equation (6) as long as the ratio of f_1_/f_3_ remains unchanged. This observation brings up the essence of labeling balance equations. The labeling balance equations describe the ratio of converging fluxes.

Similar to the FBP labeling equations, based on the labeling balance of DHAP and GAP we have the following equations

(7) $$\begin{array}{c} \left[\begin{array}{c} \begin{array}{cc} \begin{array}{c} \mathrm{FBP}{\left[1\text{-}3\right]}_{M+0}-{\mathrm{DHAP}}_{M+0}\\ \mathrm{FBP}{\left[1\text{-}3\right]}_{M+2}-{\mathrm{DHAP}}_{M+2} \end{array} & \begin{array}{c} {\mathrm{GAP}}_{M+0}-{\mathrm{DHAP}}_{M+0}\\ {\mathrm{GAP}}_{M+2}-{\mathrm{DHAP}}_{M+2} \end{array} \end{array} \end{array}\right]\left[\begin{array}{c} f_{2}\\ f_{5} \end{array}\right]=0 \end{array}$$

(8) $$\begin{array}{c} \left[\begin{array}{c} \begin{array}{cc} \begin{array}{c} \mathrm{FBP}{\left[4\text{-}6\right]}_{M+0}-{\mathrm{GAP}}_{M+0}\\ \mathrm{FBP}{\left[4\text{-}6\right]}_{M+2}-{\mathrm{GAP}}_{M+2} \end{array} & \begin{array}{c} {\mathrm{DHAP}}_{M+0}-{\mathrm{GAP}}_{M+0}\\ {\mathrm{DHAP}}_{M+2}-{\mathrm{GAP}}_{M+2} \end{array} \end{array} \end{array}\right]\left[\begin{array}{c} f_{2}\\ f_{4} \end{array}\right]=0 \end{array}$$

In the above equations, FBP[1-3] and FBP[4-6] denote the labeling patterns of the first and the last three carbon atoms of FBP, which are converted to DHAP and GAP, respectively, by the aldolase reaction. It is noteworthy that FBP[1-3] and FBP[4-6] are used only to calculate DHAP and GAP. The labeling of FBP[1-6] is not the convolution of FBP[1-3] and FBP[4-6]. The labeling balance of DHAP provides us the ratio between f_2_ and f_5_, which are the two fluxes making DHAP. The labeling balance of GAP provides us with the ratio between f_2_ and f_4_, which are the two fluxes making GAP. Equations (6)–(8) can be combined as

(9) $$\begin{array}{c} \;\left[\begin{array}{c} \begin{array}{ccccc} \begin{array}{c} {\mathrm{Glc}}_{M+0}-{\mathrm{FBP}}_{M+0}\\ {\mathrm{Glc}}_{M+2}-{\mathrm{FBP}}_{M+2}\\ {\mathrm{Glc}}_{M+4}-{\mathrm{FBP}}_{M+4} \end{array} & \begin{array}{c} 0\\ 0\\ 0 \end{array} & \begin{array}{c} {\left(\mathrm{DHAP}⊕\mathrm{GAP}\right)}_{M+0}-{\mathrm{FBP}}_{M+0}\\ {\left(\mathrm{DHAP}⊕\mathrm{GAP}\right)}_{M+2}-{\mathrm{FBP}}_{M+2}\\ {\left(\mathrm{DHAP}⊕\mathrm{GAP}\right)}_{M+4}-{\mathrm{FBP}}_{M+4} \end{array} & \begin{array}{c} 0\\ 0\\ 0 \end{array} & \begin{array}{c} 0\\ 0\\ 0 \end{array}\\ \begin{array}{c} 0\\ 0 \end{array} & \begin{array}{c} \mathrm{FBP}{\left[1\text{-}3\right]}_{M+0}-{\mathrm{DHAP}}_{M+0}\\ \mathrm{FBP}{\left[1\text{-}3\right]}_{M+2}-{\mathrm{DHAP}}_{M+2} \end{array} & \begin{array}{c} 0\\ 0 \end{array} & \begin{array}{c} 0\\ 0 \end{array} & \begin{array}{c} {\mathrm{GAP}}_{M+0}-{\mathrm{DHAP}}_{M+0}\\ {\mathrm{GAP}}_{M+2}-{\mathrm{DHAP}}_{M+2} \end{array}\\ \begin{array}{c} 0\\ 0 \end{array} & \begin{array}{c} \mathrm{FBP}{\left[4\text{-}6\right]}_{M+0}-{\mathrm{GAP}}_{M+0}\\ \mathrm{FBP}{\left[4\text{-}6\right]}_{M+2}-{\mathrm{GAP}}_{M+2} \end{array} & \begin{array}{c} 0\\ 0 \end{array} & \begin{array}{c} {\mathrm{DHAP}}_{M+0}-{\mathrm{GAP}}_{M+0}\\ {\mathrm{DHAP}}_{M+2}-{\mathrm{GAP}}_{M+2} \end{array} & \begin{array}{c} 0\\ 0 \end{array} \end{array} \end{array}\right]\left[\begin{array}{c} f_{1}\\ f_{2}\\ f_{3}\\ f_{4}\\ f_{5} \end{array}\right]=0 \end{array}$$

Note that Equation (9) is a set of homogeneous linear equations. All fluxes are unitless and can be scaled up or down without violating the equation. Only when the absolute value of f_1_ is measured by other experiments, these fluxes can be expressed in absolute values as well.

The general form of the labeling balance equation should be apparent by now. Suppose we have n fluxes making the same metabolite, which has the labeling pattern of $\bar{m}$. If each substrate i has the labeling pattern of $m_{i}$ and is contributing to the product through flux *f_i_*, then

(10) $$\begin{array}{c} \overset{n}{\underset{i=1}{\sum }}f_{i}\left(m_{i}-\bar{m}\right)=0 \end{array}$$

Equation (10) illustrates several fundamental principles of MFA. First, the use of isotope labeling is required to solve fluxes. While this statement seems obvious, it naturally emerges from Equation (10). If no tracer is used, then the labeling pattern of all substrates ($m_{i}$) equals to that of the product ($\bar{m}$). Therefore, the matrix $\left(m_{i}-\bar{m}\right)$ contains only zero vectors and $f_{i}$ can take any value to satisfy the equation. For the same reason, if the two substrates of one metabolite are identically labeled (e.g., when the U-^13^C glucose tracer is used, all metabolites would be fully labelled with ^13^C), the fluxes from these substrates still cannot be solved. This is the principle guiding the tracer selection in MFA, which will be discussed later in more detail. Second, at the isotope labeling steady state, the labeling pattern of a metabolite is determined only by its production fluxes and is independent of its consumption fluxes. The consumption flux of one metabolite shows up in the labeling balance equations only when it becomes the production flux of another metabolite. Third, metabolite concentration is not in this equation, meaning that under isotopic steady state the fluxes are independent of the metabolite pool size. This is the concept we discussed in the introduction. It is also a natural consequence of the steady-state labeling balance equation. We will show later that under the isotopic non-steady state condition the metabolite pool size is related with fluxes.

## 3. Generalization of the Labeling Balance Equations

In order to calculate the labeling of DHAP and GAP, we introduced the terms FBP[1-3] and FBP[4-6] in Equations (7) and (8), which are parts of the FBP molecule. These terms are commonly referred to as elementary metabolite units (EMU). EMU is defined as a moiety comprising any distinct subset of a compound’s atoms [66]. The use of EMU is a natural choice when calculating the labeling pattern of metabolites synthesized by combining the carbon backbones. EMU significantly simplifies the flux calculation. Taking FBP as an example, there are totally 2^6^ = 64 different forms in which FBP can be ^13^C-labeled. Using the EMU approach, we are only concerned with three EMUs for FBP: FBP[1-3], FBP[4-6] and FBP[1-6]. Without this simplification process, the calculation of fluxes would become much more difficult. For example, if all the possible EMU should be described, Equation (3) would have to use 128 unknown parameters to describe the labeling of DHAP⊕GAP and FBP, and 64 known parameters to describe the labeling of glucose tracer. The overall workload becomes unnecessarily large.

EMU should also account for the molecular symmetry in the metabolites. In citric acid cycle, for example, succinate is a symmetric molecule. The first and fourth carbon of succinate (Suc [1] and Suc [4]) should always have the same labeling pattern. A sufficiently large “flipping” flux that interconverts Suc [1234] and Suc [4321] can be introduced to the flux model to account for such symmetry. It is also noteworthy that such flipping flux should not be applied to pro-chiral metabolites. Prochirality of a metabolite is often overlooked. In fact, prochirality is easy to identify in the isotope labeling experiment. A molecule is pro-chiral if isotope labeling can convert it from achiral to chiral. Citrate, for example, has two -CH_2_COOH groups, one derived from acetyl-CoA and the other one from oxaloacetate. If the acetyl-CoA is ^13^C-labeled, the ^13^C_2_-citrate becomes a chiral molecule. This suggests that citrate is a pro-chiral molecule. Therefore, even though the two -CH_2_COOH groups are symmetric with respect to a mirror plane, they are not interconvertible.

EMU is the natural choice to describe the labeling pattern of metabolites when they are measured in the forms of mass isotopomer distributions (MIDs) by mass spectrometry. However, if NMR instead of MS is used, the labeling pattern would be described more easily in the form of positional labeling. One such approach is known as cumomers [67]. The cumomer approach is only concerned with the percentage that FBP and other metabolites that are labeled at specific carbon atoms, regardless of the labeling at other positions. In this case, we can rewrite Equations (6)–(8), which describes the labeling balance in EMU terms, using the cumomer approach as the following.

(11) $$\begin{array}{c} \left[\begin{array}{cc} \begin{array}{c} \mathrm{Glc}[2]-\mathrm{FBP}[2]\\ \mathrm{Glc}[5]-\mathrm{FBP}[5] \end{array} & \begin{array}{c} \mathrm{DHAP}[2]-\mathrm{FBP}[2]\\ \mathrm{GAP}[2]-\mathrm{FBP}[5] \end{array} \end{array}\right]\left[\begin{array}{c} f_{1}\\ f_{3} \end{array}\right]=0 \end{array}$$

(12) $$\left[\begin{array}{cc} \mathrm{FBP}[2]-\mathrm{DHAP}[2] & \mathrm{GAP}[2]-\mathrm{DHAP}[2] \end{array}\right]\left[\begin{array}{c} f_{2}\\ f_{5} \end{array}\right]=0$$

(13) $$\left[\begin{array}{cc} \mathrm{FBP}[5]-\mathrm{GAP}[2] & \mathrm{DHAP}[2]-\mathrm{GAP}[2] \end{array}\right]\left[\begin{array}{c} f_{2}\\ f_{4} \end{array}\right]=0$$

In these equations, we are concerned with FBP labeling on C-2 and C-5 position, which is FBP $010000 and FBP $000010 in the cumomer terms. This example demonstrates that EMUs and cumomers follow exactly the same labeling balance equations. They are only different in how the labeling patterns are expressed.

Another way to express the isotope labeling is by enrichment. Enrichment is defined as the percentage of labeling on average atom level. The enrichment of FBP (E_FBP_) can be calculated from its labeling pattern.

(14) $$\begin{array}{c} E_{\mathrm{FBP}}={\left[\begin{array}{c} 0/6\\ 1/6\\ 2/6\\ 3/6\\ 4/6\\ 5/6\\ 6/6 \end{array}\right]}^{T}\left[\begin{array}{c} {\mathrm{FBP}}_{M+0}\\ {\mathrm{FBP}}_{M+1}\\ {\mathrm{FBP}}_{M+2}\\ {\mathrm{FBP}}_{M+3}\\ {\mathrm{FBP}}_{M+4}\\ {\mathrm{FBP}}_{M+5}\\ {\mathrm{FBP}}_{M+6} \end{array}\right]\times 100\% \end{array}$$

The good property of enrichment is that the enrichment of the product in a combination reaction is the carbon number-weighted average of the enrichment of the substrates.

(15) $$\begin{array}{c} E_{\mathrm{DHAP}⊕\mathrm{GAP}}=\left(E_{\mathrm{DHAP}}\times 3+E_{\mathrm{GAP}}\times 3\right)/6\times 100\% \end{array}$$

Isotopic enrichment is commonly used for radioactive tracers. For stable isotope tracers, this is also a convenient term because the labeling vector is converted to a single number. Nonetheless, the labeling balance follows Equation (10) when using the enrichment to represent the labeling patterns.

## 4. Basic Assumptions in MFA

Having shown that the labeling balance Equation (10) is the basis for many different versions of MFA, it is time to examine the basic assumptions behind Equation (10) and MFA. The first assumption that we have adopted in this equation is that the fluxes are linear with respect to all the labeled fractions. This means the enzymes and transporters do not discriminate between unlabeled and labeled substrates. In another word, there is no kinetic isotope effect (KIE) [68,69,70,71,72]. As Liuni et al. [69] has shown, ^12^C/^13^C KIE is typically < 1.1 so this assumption is largely valid in carbon labeling experiments. In deuterium (^2^H)-labeling experiments, however, strong KIE (>2–4) may come in play. The KIE makes the subtraction of a ^2^H atom slower than the subtraction of a ^1^H atom. This rate difference can be measured using isolated enzymes and substrates in a test tube. However, the cellular homeostatic mechanisms may result in a smaller impact on the isotope labelling patterns in cells [73]. One possibility is that the total metabolic fluxes are maintained even when the heavy isotope tracers are used. This is likely the case when the enzyme has remaining capacity at the physiological flux level. Another possibility is that the pathway flux is decreased by the same magnitude that the enzyme reaction in vitro is slowed down due to the heavy-labeled substrate. The actual flux change due to KIE is likely somewhere in between and should be evaluated on a case-by-case basis. To introduce KIE to the labeling balance equation of MFA, the flux coefficients ($f_{i}$; Equation (10)) should be replaced by row vectors of fluxes specific to each labeled fraction.

The second assumption adopted in Equation (10) is that all the metabolites are well-mixed. We are assuming that each metabolite is labeled homogenously so that its labeling pattern can be expressed in a single vector. This simple assumption has different meanings and implications on different levels in the biological system. On the molecular level, the intermediates in a metabolic pathway can be passed from one enzyme to the next without equilibration with the cellular pool [74]. This process is known as substrate channeling [75,76,77]. The assumption that the metabolites are well-mixed means there is no substrate channeling. On the cellular level, a metabolite can be distributed in different cellular compartments, such as cytosol and mitochondrion [78,79,80]. The well-mixed assumption means either the metabolite is exclusively in one cellular compartment, or there is fast exchange among the compartments so that the labeling patterns are averaged out. On the organ level, we have a mixture of different types of cells. We are assuming these cells share the same labeling patterns of metabolites, or there is fast exchange among cells. When these well-mixed assumptions are violated, separate pools of metabolites should be considered when designing MFA models to address the compartmentalization or substrate channeling issues.

## 5. Predicting Labeling Patterns and Solving Metabolic Fluxes

Now we are ready to solve the fluxes for our example of upper glycolysis pathway. Since the value of f_1_ is measured as 100 nmol/h, our goal is to find a set of fluxes f_2_–f_6_ that generates a labeling pattern of FBP that best matches the observation. Even though the labeling patterns of DHAP and GAP are not measurable, their labeling patterns can still be predicted along with that of FBP when a set of fluxes are given. Based on the mass balance on each of the labeled fractions of DHAP, GAP and FBP, we have the following equations.

(16) $$\begin{array}{c} \{\begin{array}{c} f_{2}\times FBP\left[1\text{-}3\right]+f_{5}\times GAP=\left(f_{2}+f_{5}\right)\times DHAP\\ f_{2}\times FBP\left[4\text{-}6\right]+f_{4}\times DHAP=\left(f_{2}+f_{4}\right)\times GAP\\ f_{3}\times DHAP+f_{1}\times Glc\left[1\text{-}3\right]=(f_{3}+f_{1})\times FBP\left[1\text{-}3\right]\\ f_{3}\times GAP+f_{1}\times Glc\left[4\text{-}6\right]=(f_{3}+f_{1})\times FBP\left[4\text{-}6\right] \end{array} \end{array}$$

Due to the mass balance of FBP, DHAP and GAP, we have another set of equations.

(17) $$\begin{array}{c} \{\begin{array}{c} f_{1}+f_{3}=f_{2}\\ f_{2}+f_{5}=f_{3}+f_{4}\\ f_{2}+f_{4}=f_{3}+f_{5}+f_{6} \end{array} \end{array}$$

Since f_1_ = 100. There are only two free fluxes f_3_ and f_5_. All other fluxes can be calculated from these two free fluxes because of the mass balance.

(18) $$\begin{array}{c} \{\begin{array}{c} f_{2}=100+f_{3}\\ f_{4}=100+f_{5}\\ f_{6}=200 \end{array} \end{array}$$

Take Equation (18) into Equation (16), we have the following equation.

(19) $$\begin{array}{c} (100+f_{3})\times FBP\left[1\text{-}3\right]+f_{5}\times GAP=\left(100+f_{3}+f_{5}\right)\times DHAP\\ \left(100+f_{3}\right)\times FBP\left[4\text{-}6\right]+(100+f_{5})\times DHAP=\left(200+f_{3}+f_{5}\right)\times GAP\\ f_{3}\times DHAP+100\times Glc\left[1\text{-}3\right]=\left(100+f_{3}\right)\times FBP\left[1\text{-}3\right]\\ f_{3}\times GAP+100\times Glc\left[4\text{-}6\right]=\left(100+f_{3}\right)\times FBP\left[4\text{-}6\right] \end{array}$$

Equation (19) illustrates how we can calculate labeling patterns from the free fluxes. For example, if we take the values of f_3_ = 50 and f_5_ = 150, the four unknowns of FBP[1-3], FBP[4-6], DHAP and GAP become solvable through Equation (20). Since we used the tracer of 1,2-^13^C_2_-glucose, $\mathrm{Glc}{\left[1\text{-}3\right]}_{M+2}$ and $\mathrm{Glc}{\left[4\text{-}6\right]}_{M+0}$ are 1. All other forms of Glc[1-3] and Glc[4-6] are 0.

(20) $$\begin{array}{c} \begin{array}{c} \left[\begin{array}{cccc} 150 & 0 & -300 & 150\\ 0 & 150 & 250 & -400\\ -150 & 0 & 50 & 0\\ 0 & -150 & 0 & 50 \end{array}\right]\left[\begin{array}{c} \begin{array}{c} \begin{array}{c} \mathrm{FBP}\left[1\text{-}3\right]\\ \mathrm{FBP}\left[4\text{-}6\right] \end{array}\\ \mathrm{DHAP}\\ \mathrm{GAP} \end{array} \end{array}\right]=\left[\begin{array}{cc} 0 & 0\\ 0 & 0\\ -100 & 0\\ 0 & -100 \end{array}\right]\left[\begin{array}{c} \begin{array}{c} \mathrm{Glc}\left[1\text{-}3\right]\\ \mathrm{Glc}\left[4\text{-}6\right] \end{array} \end{array}\right] \end{array}\\ \begin{array}{c} \;=\left[\begin{array}{cc} 0 & 0\\ 0 & 0\\ -100 & 0\\ 0 & -100 \end{array}\right]\left[\begin{array}{c} \begin{array}{cc} \mathrm{Glc}{\left[1\text{-}3\right]}_{M+0} & \mathrm{Glc}{\left[1\text{-}3\right]}_{M+2}\\ \mathrm{Glc}{\left[4\text{-}6\right]}_{M+0} & \mathrm{Glc}{\left[4\text{-}6\right]}_{M+2} \end{array} \end{array}\right] \end{array}\\ \begin{array}{c} \;\;=\left[\begin{array}{cc} 0 & 0\\ 0 & 0\\ -100 & 0\\ 0 & -100 \end{array}\right]\left[\begin{array}{c} \begin{array}{cc} 0 & 1\\ 1 & 0 \end{array} \end{array}\right] \end{array} \end{array}$$

To solve the FBP[1-3], FBP[4-6], DHAP and GAP, we simply need to take the inverse of the left matrix containing the fluxes and multiply it with the other two matrices on the right.

(21) $$\begin{array}{c} \begin{array}{c} \left[\begin{array}{c} \begin{array}{c} \begin{array}{c} \mathrm{FBP}\left[1\text{-}3\right]\\ \mathrm{FBP}\left[4\text{-}6\right] \end{array}\\ \mathrm{DHAP}\\ \mathrm{GAP} \end{array} \end{array}\right]=\left[\begin{array}{c} \begin{array}{cc} \begin{array}{c} \mathrm{FBP}{\left[1\text{-}3\right]}_{M+0}\\ \mathrm{FBP}{\left[4\text{-}6\right]}_{M+0} \end{array} & \begin{array}{c} \mathrm{FBP}{\left[1\text{-}3\right]}_{M+2}\\ \mathrm{FBP}{\left[4\text{-}6\right]}_{M+2} \end{array}\\ {\mathrm{DHAP}}_{M+0} & {\mathrm{DHAP}}_{M+2}\\ {\mathrm{GAP}}_{M+0} & {\mathrm{GAP}}_{M+2} \end{array} \end{array}\right] \end{array}\\ \begin{array}{c} =\;{\left[\begin{array}{cccc} 150 & 0 & -300 & 150\\ 0 & 150 & 250 & -400\\ -150 & 0 & 50 & 0\\ 0 & -150 & 0 & 50 \end{array}\right]}^{-1}\left[\begin{array}{cc} 0 & 0\\ 0 & 0\\ -100 & 0\\ 0 & -100 \end{array}\right]\left[\begin{array}{c} \begin{array}{cc} 0 & 1\\ 1 & 0 \end{array} \end{array}\right] \end{array}\\ \begin{array}{c} \;=\left[\begin{array}{cc} 0.1000 & 0.9000\\ 0.8333 & 0.1667\\ 0.3000 & 0.7000\\ 0.5000 & 0.5000 \end{array}\right] \end{array} \end{array}$$

Therefore, we can calculate FBP labeling from Equation (3).

(22) $$\begin{array}{c} \begin{array}{c} \mathrm{FBP}=[f_{3}\times \left(DHAP⊕GAP\right)+f_{1}\times Glc]/f_{2} \end{array}\\ \begin{array}{c} =f_{3}/f_{2}\times \left[\begin{array}{c} {\left(\mathrm{DHAP}⊕\mathrm{GAP}\right)}_{M+0}\\ {\left(\mathrm{DHAP}⊕\mathrm{GAP}\right)}_{M+2}\\ {\left(\mathrm{DHAP}⊕\mathrm{GAP}\right)}_{M+4} \end{array}\right]+f_{1}/f_{2}\times \left[\begin{array}{c} {\mathrm{Glc}}_{M+0}\\ {\mathrm{Glc}}_{M+2}\\ {\mathrm{Glc}}_{M+4} \end{array}\right] \end{array}\\ \begin{array}{c} =\frac{f_{3}}{f_{2}}\times \left[\begin{array}{c} 0.1500\\ 0.5000\\ 0.3500 \end{array}\right]+\frac{f_{1}}{f_{2}}\times \left[\begin{array}{c} 0.0000\\ 1.0000\\ 0.0000 \end{array}\right]=\left[\begin{array}{c} 0.0500\\ 0.8333\\ 0.1167 \end{array}\right] \end{array} \end{array}$$

**Figure 2:**
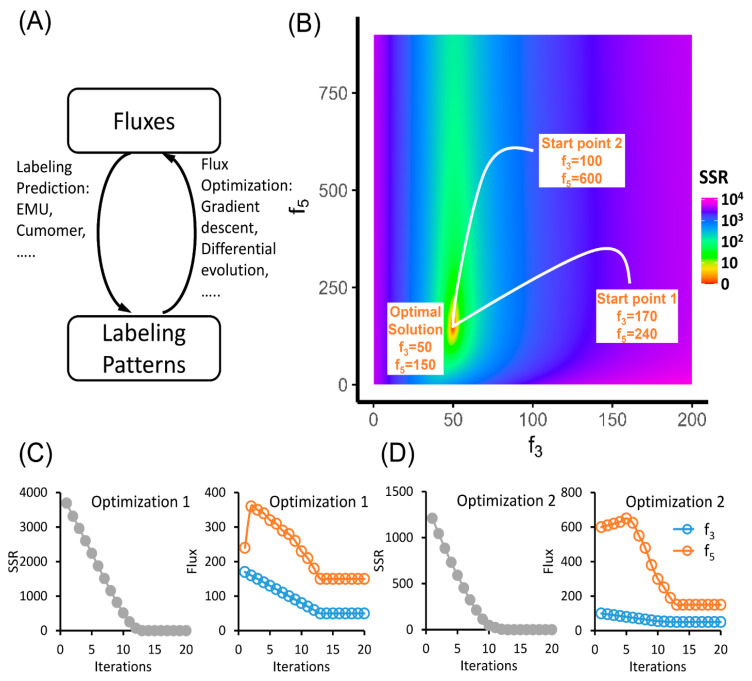
The solving process of MFA. (**A**) The relationship between fluxes and labeling patterns. The choice of tracer and fluxes can determine the labeling patterns, which can be calculated by elementary metabolite units (EMU), cumomer or other methods. The flux-solving process is the inverse problem of the labeling prediction, which involves iterative fitting between the labeling pattern predicted from “guessed” fluxes and the observed labeling pattern. (**B**–**D**) Iterative paths of optimization from two different start points ending with the same solution. (**B**) The landscape plot of sum of squared residuals (SSR) on the plane of free fluxes. Each dot on this plane represents a combination of fluxes, which gives a prediction on the labeling pattern of FBP. This predicted labeling pattern is compared to the measurement and the discrepancy between them is defined as the SSR and is plotted with different colors on the plane. For flux calculation, we can start from a random point and perform gradient descent or other optimization algorithms to minimize the SSR. The iterative paths starting from two randomly chosen points are shown. (**C**,**D**) Change of SSR and fluxes from start point 1 (**C**) and 2 (**D**) to the optimal solution.

Therefore, by given the values of two free fluxes f_3_ = 50 and f_5_ = 150, the labeling pattern of FBP can be predicted as 5.00% M+0, 83.33% M+2 and 11.67% M+4. The real MFA process, however, is the reverse of this question (Figure 2A): if the actual labeling pattern of FBP is given, can we find the value of f_3_ and f_5_ that best fit this observation? Since in most cases there is no direct way to calculate the free fluxes by simply plugging in the numbers, the best-fit fluxes are often solved as an optimization problem. The flux-solving process is essentially a “guessing game”, which is iterative and consists of two steps: (1) calculate the labeling patterns of FBP based on the “guessed flux”, and (2) update the “guessed flux” to reduce the discrepancy between the predicted FBP labeling pattern and the observation. The discrepancy (R) can be defined as the sum of squared residuals (SSR), and it is often inversely weighted by the experimental standard deviations expressed as the measurement covariance matrix with measurement variances located on the diagonal (Σ_Measured_) [81].

(23) $$\begin{array}{c} R={\left({\left[\begin{array}{c} {\mathrm{FBP}}_{M+0}\\ {\mathrm{FBP}}_{M+2}\\ {\mathrm{FBP}}_{M+4} \end{array}\right]}_{\mathrm{Measured}}-{\left[\begin{array}{c} {\mathrm{FBP}}_{M+0}\\ {\mathrm{FBP}}_{M+2}\\ {\mathrm{FBP}}_{M+4} \end{array}\right]}_{\mathrm{Prediction}}\right)}^{T}\Sigma_{\mathrm{Measured}}^{-1}\left({\left[\begin{array}{c} {\mathrm{FBP}}_{M+0}\\ {\mathrm{FBP}}_{M+2}\\ {\mathrm{FBP}}_{M+4} \end{array}\right]}_{\mathrm{Measured}}-{\left[\begin{array}{c} {\mathrm{FBP}}_{M+0}\\ {\mathrm{FBP}}_{M+2}\\ {\mathrm{FBP}}_{M+4} \end{array}\right]}_{\mathrm{Prediction}}\right)\\ =\underset{i=0,2,4}{\sum }{\left(\frac{{\mathrm{FBP}}_{\mathrm{Measured}}^{M+i}-{\mathrm{FBP}}_{\mathrm{Prediction}}^{M+i}}{\mathrm{SD}\left({\mathrm{FBP}}_{\mathrm{Measured}}^{M+i}\right)}\right)}^{2} \end{array}$$

Once the discrepancy is sufficiently small or no better guesses can be made, the optimized flux combination is our best estimation. Figure 2B–D demonstrated such a process. By starting from a random flux combination, the gradient descent algorithm can find the direction to minimize the discrepancy between the labeling measurements and the model prediction. When the discrepancy cannot be further minimized, the optimization completes, and the optimal flux combination is the solution.

From this example, we can see that the calculation of the labeling patterns is the most important part in the flux determination. The labeling calculation gives us the landscape, such as shown in Figure 2B, which describes the difference between the model prediction and the experimental measurements when the flux combination takes different values. The goal of the optimization is to find the path on the landscape to reach the lowest point. There are many generic algorithms that can work for such a purpose, including gradient descent, differential evolution and particle swarm optimization algorithms.

## 6. Evaluation of MFA Result and Tracer Selection

In the example of upper glycolysis, if we start from the FBP measurement of 5.00% M+0, 83.33% M+2 and 11.67% M+4, we can land on the flux combination of f_3_ = 50 and f_5_ = 150. This solution is both unique and precise. No other flux combination can generate exactly the same FBP labeling pattern. However, in the real experiments, because of the existence of measurement errors, we may not have a flux combination that perfectly fit the measurements. The optimization algorithms can help us to achieve a locally best solution. However, it is hard to know whether this is the best situation globally. One workaround is to run the optimization algorithm multiple times with a random starting point in each round. If the solutions converge to one flux combination, we have confidence that this is the globally best solution (Figure 2B). If multiple solutions are reported, we can compare the results and choose the best one.

**Figure 3:**
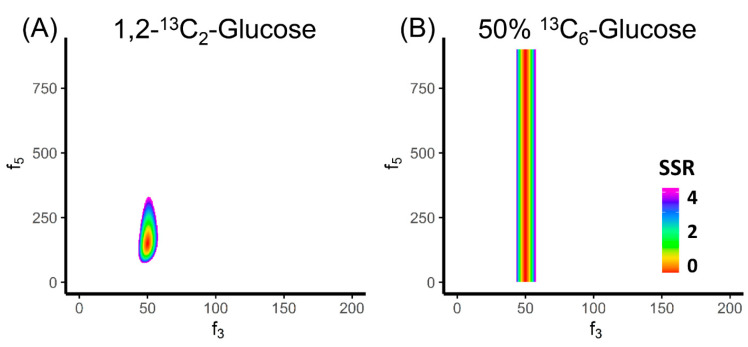
Evaluation on the tracer selection for MFA. (**A**) The colored region is showing the 95% confidence region of f_3_ and f_5_ using 1,2-^13^C_2_ glucose as tracer, assuming 1% error on all labeled fractions of FBP. Both f_3_ and f_5_ can be relatively well-determined. (**B**) The colored region is showing the 95% confidence region of f_3_ and f_5_ using 50% U-^13^C- glucose + 50% unlabeled glucose as the tracer. Only f_3_ can be determined.

In addition to the best flux combination, we want to know the uncertainty in each flux to fully evaluate the results. The flux uncertainty is commonly represented as the confidence intervals of individual fluxes. When the flux takes a sub-optimal value within the confidence interval, the SSR goes up but is still within the statistically acceptable range. For example, if we assume the measurement of the MID of FBP has 1% error, then the combination of f_3_ = 48 and f_5_ = 210 would also be an acceptable solution since its SSR (χ^2^ = 1.33) was lower than the threshold assuming 1% error on all labeled fractions (χ^2^(α = 0.95,df = 1) = 3.84) [81]. In fact, if we plot all the acceptable solutions in the coordinate system of f_3_ and f_5_, an area of confidence interval is generated (Figure 3A). In this case, the area of confidence is in an oval shape ranging from 43.5 to 57.5 and from 75 to 330 on the x and y axis, respectively. These ranges are also the confidence intervals for f_3_ and f_5_. A narrow confidence interval on a flux indicates that the SSR is sensitive to the change of this flux. Therefore, this flux can be determined more precisely. On the other hand, a wide confidence interval suggests the SSR is insensitive to the change of this flux and the flux is poorly determined. It should be noted that the range of the confidence interval is determined by the precision of the MID measurements and the sensitivity of the SSR to the fluxes. The flux sensitivity is a local property. When the optimal flux fit changes, the flux confidence interval should be re-evaluated.

By drawing the area of confidence intervals, we can also evaluate the tracer selection for MFA. The choice of the tracer has tremendous impact on the effectiveness of MFA. For example, 1,2-^13^C_2_ glucose is an effective tracer to determine the free fluxes f_3_ and f_5_ in the upper glycolysis network (Figure 3A). In contrast, if we use 50% U-^13^C- glucose + 50% unlabeled glucose, we can only determine f_3_ but not f_5_ (Figure 3B). This observation motivates us to investigate the principle behind tracer selection to achieve the best effectiveness of MFA.

Essentially, the principle for tracer selection is illustrated in Equation (10). The role of the tracer in MFA is to make the metabolite labeling patterns reflective of the fluxes. More specifically, if one metabolite can be made by two or more reactions, the labeling pattern of this metabolite is the flux-weighted average of the labeling of the substrates. This allows us to determine the ratio of the converging fluxes. However, if the fluxes of two reactions utilize substrates with the same labeling pattern, the product can be only made in the same labeling pattern, and no flux ratio can be determined. In the example of upper glycolysis, when 50% U-^13^C glucose and 50% unlabeled glucose was used as the tracer, the labeling patterns of FBP[1-3] and GAP became identical (50% M+0; 50% M+3). Therefore, no matter how f_5_ changes, the labeling pattern of DHAP would remain the same. In another word, the labeling pattern of DHAP can no longer reflect the ratio between f_3_ and f_5_. Thus, the confidence region of the free fluxes is a vertical belt (Figure 3B). In contrast, the value of f_3_ can still be determined. This is because the labeling patterns of the two substrates glucose and DHAP⊕GAP are still different. On the network level, we can assess tracer effectiveness by calculating the sensitivity. The good tracer makes the labeling patterns sensitive to the changes of the flux inputs. Multiple studies have been conducted to evaluate the performance of different tracers on evaluating fluxes in glycolysis [82,83], the pentose phosphate pathway [83,84,85] and the TCA cycle [83,86].

**Figure 4:**
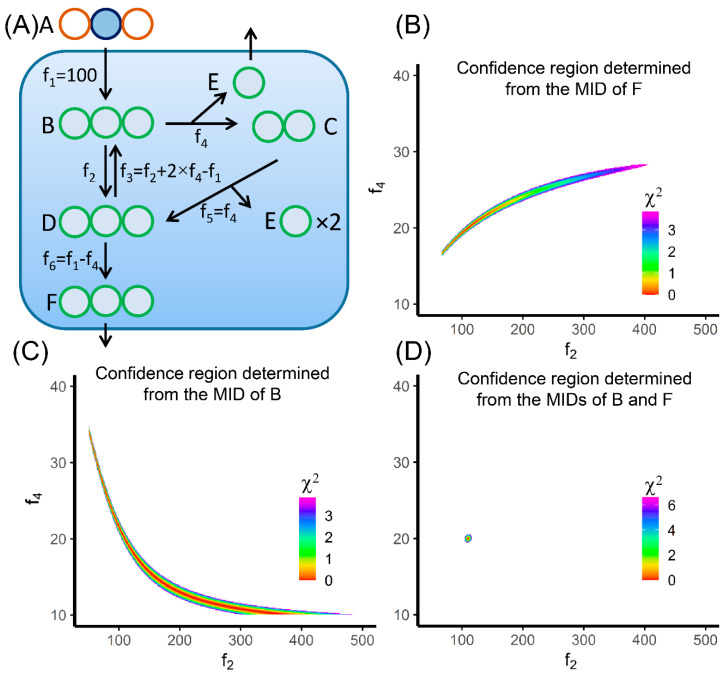
Plotting flux confidence region for the network from Antoniewicz et al. [66]. (**A**) The network structure. f_2_ and f_4_ are two free fluxes in this model network. 2-^13^C_1_ A is used as a tracer. (**B**) The colored region is showing the 95% confidence region determined by measuring the labeling pattern of F only. (**C**) The colored region is showing the 95% confidence region determined by measuring the labeling pattern of B only. (**D**) The colored region is showing the 99% confidence region, in order to enlarge the region to be more visible, determined by measuring the labeling patterns of both B and F. The discrepancy between predicted labeling pattern and the measurement is converted to the χ^2^-statistic assuming 0.3% error on the labeled fractions.

It should be noted that the confidence interval of each flux is not independent from others in the more general cases. In Figure 4, we are demonstrating the flux confidence intervals for the metabolic network presented in the EMU paper [66]. f_2_ and f_4_ are the two free fluxes in this model (Figure 4A). We are using 2-^13^C_1_-A as the tracer, and the MID measurement is made on metabolite F. If we allow 0.3% error on each of the labeled fractions, the confidence intervals of f_2_ and f_4_ are 16.4-28.3 and 67–405, respectively. The overall confidence region plotted on the f_2_–f_4_ plane is a belt from lower left to the upper right (Figure 4B). The direction of the belt means when f_2_ gets larger, f_4_ needs to go larger to minimize the increase of the SSR. The confidence intervals of these two free fluxes are highly correlated. The wide confidence intervals of f_2_ and f_4_ suggest the fluxes are poorly determined when given the MID measurement of F. This result is seemingly odd because we only have two free fluxes to determine, yet four numbers were measured (M+0–M+3) from the MID of F. The reason that these fluxes are still poorly determined is because the four measurements from the MID of F are not totally independent. Similarly, if we only measure the MID of B in this metabolic network, we also have wide confidence intervals on f_2_ and f_4_ (Figure 4C). In this case, these two fluxes are anti-correlated. When f_2_ gets larger, f_4_ needs to go smaller to minimize the increase of the SSR. This observation suggests that we need to measure the MIDs of both B and F to achieve best precision of MFA (Figure 4D). This example demonstrates that the identifiability of flux should be assessed seriously. Simply having more fractional numbers in the MIDs than the number of free fluxes does not guarantee identifiability [87].

Figure 4B,C also show that the confidence region is non-linear and non-convex. Therefore, the calculation of confidence intervals is not quite straightforward, especially in large metabolic network with a large number of free fluxes. The method to accurately calculate confidence intervals has been proposed by Antoniewicz et al. [81]. In brief, the confidence intervals were calculated by (1) moving one target flux away from the best-fit value by a small step; (2) choosing a combination of the other fluxes that minimize the increase of SSR; and (3) calculating the new SSR and repeating step (1) to (3) until the new SSR reached the cutoff for 95% confidence interval. An alternative is the Markov Chain Monte Carlo (MCMC) method. We could sample in the flux space and allow the MID and other measurements to have Gaussian error terms. The distribution of the flux values from the perturbed measurements is used to approximate the flux confidence intervals. The advantage of MCMC method is that it can generate a comprehensive probability distribution of fluxes [88,89].

## 7. Kinetic Flux Profiling and Isotopically Non-Stationary MFA

So far, all the MFA we have discussed are based on the assumptions of steady states. Essentially, the assumptions are (1) the metabolic steady state is the condition that the metabolite pool sizes and fluxes do not change over time and (2) isotopic steady state is the condition that the labeling patterns of metabolites do not change with time [90]. Experimentally, the isotopic steady state is achieved by incubating or infusing the tracer for sufficiently long time so that the labeling patterns of the metabolites do not change anymore. Under this condition, the ratio of the converging fluxes can be determined if the substrates are labeled in different patterns. Moreover, the size of metabolite pools does not affect the labeling patterns. One limitation of isotopically stationary MFA is that it does not work on pathways where no differential labeling can be observed. For example, CO_2_ is the sole carbon source of the photosynthetic product in autotrophic tissues of plant. At isotopic steady state, all the photosynthetic intermediates and products would have exactly the same labeling as CO_2_. Consequently, no flux information can be derived [91]. One solution to such a problem is kinetic flux profiling (KFP) [92]. In KFP experiments, we can switch the carbon source from unlabeled to the labeled one and examine the labeling patterns of intermediates and products at different time points. The key idea in KFP is that turnover rate of the metabolite pool is determined by the synthesizing flux and the pool size of metabolite. In fact, KFP is the extension of MFA in isotopic non-steady state case. In the final part of this review paper, we wish to highlight the link between steady state MFA and KFP in isotopic non-steady state [92,93,94,95], and further extend it to the general non-steady state case.

For an intracellular metabolite under isotopic non-steady state, the total pool labeling change with regard to all the labeled fractions can be expressed as

(24) $$\begin{array}{c} \Delta \bar{M}=\overset{n}{\underset{i=1}{\sum }}\left(\int_{0}^{T}f_{i}m_{i}Vdt\right)-\int_{0}^{T}f_{out}\bar{m}Vdt \end{array}$$

$\Delta \bar{M}$ represents the net change in a metabolite from *t* = 0 to *t* = *T*. $\Delta \bar{M}$ can be the amount of a specific labeled fraction, or a vector to describe all labeled fractions. $f_{i}$ represents the production flux from the *i*-th substrate. $f_{out}$ represents the sum of the consumption fluxes. These fluxes are normalized to cell volume *V*. $m_{i}$ represents the labeling pattern of the *i*-th substrate and $\bar{m}$ represents the labeling pattern of the product. When expressed in vector forms, the $\Delta \bar{M}$, $m_{i}$ and $\bar{m}$ describe the same set of labeled fractions.

Under metabolic steady state, where fluxes and the cell volume are constant over time, and the consumption flux equals the sum of the production fluxes, we have

(25) $$\begin{array}{c} \Delta \bar{M}=\overset{n}{\underset{i=1}{\sum }}\left(f_{i}V\int_{0}^{T}m_{i}dt\right)-f_{out}V\int_{0}^{T}\bar{m}dt\\ =\overset{n}{\underset{i=1}{\sum }}\left(f_{i}V\int_{0}^{T}m_{i}dt\right)-\left(\overset{n}{\underset{i=1}{\sum }}f_{i}\right)V\int_{0}^{T}\bar{m}dt\\ =\overset{n}{\underset{i=1}{\sum }}\left[f_{i}V\int_{0}^{T}\left(m_{i}-\bar{m}\right)dt\right] \end{array}$$

Furthermore, under metabolic steady state, the intracellular metabolite pool size does not change. With a constant metabolite concentration, we also have

(26) $$\begin{array}{c} \Delta \bar{M}=cV\Delta \bar{m} \end{array}$$

In Equation (26), *c* is the intracellular concentration of the metabolite and *V* is the cell volume.

Combining Equations (25) and (26), we have

(27) $$\begin{array}{c} \Delta \bar{m}=\overset{n}{\underset{i=1}{\sum }}\left[f_{i}\int_{0}^{T}\left(m_{i}-\bar{m}\right)dt\right]/c \end{array}$$

We can also take derivative of Equation (27), which gives

(28) $$\begin{array}{c} \frac{d\bar{m}}{dt}=\overset{n}{\underset{i=1}{\sum }}\left[f_{i}\left(m_{i}-\bar{m}\right)\right]/c \end{array}$$

Equation (28) is the equation describing the labeling change with respect to time for KFP. It is also the isotopic non-steady state generalization of Equation (6). When the isotopic steady state is reached, $d\bar{m}/dt$ = 0, and Equation (28) degenerates to Equation (10). This means the isotopic non-steady state and steady state cases are closely related.

Next, we assume neither isotopic steady state nor metabolic steady state. Starting from Equation (24), considering that the sum of the consumption fluxes can be replaced by the sum of production fluxes minus the net production flux, we can re-write Equation (24) to

(29) $$\begin{array}{c} \begin{array}{c} \Delta \bar{M}=\overset{n}{\underset{i=1}{\sum }}\left(\int_{0}^{T}f_{i}Vm_{i}dt\right)-\int_{0}^{T}\left(\overset{n}{\underset{i=1}{\sum }}f_{i}V-f_{Net\_Pro}V\right)\bar{m}dt \end{array}\\ \begin{array}{c} =\overset{n}{\underset{i=1}{\sum }}\left(\int_{0}^{T}f_{i}Vm_{i}dt\right)-\overset{n}{\underset{i=1}{\sum }}\left(\int_{0}^{T}f_{i}V\bar{m}dt\right)+\int_{0}^{T}f_{Net\_Pro}V\bar{m}dt \end{array}\\ \begin{array}{c} =\overset{n}{\underset{i=1}{\sum }}\left[\int_{0}^{T}f_{i}V\left(m_{i}-\bar{m}\right)dt\right]+\int_{0}^{T}f_{Net\_Pro}V\bar{m}dt \end{array} \end{array}$$

Taking the derivative of Equation (29), we have

(30) $$\begin{array}{c} \frac{d\bar{M}}{dt}=\overset{n}{\underset{i=1}{\sum }}\left[f_{i}\left(m_{i}-\bar{m}\right)V\right]+f_{Net\_Pro}\bar{m}V \end{array}$$

Without assuming constant metabolite concentration or cell volume, the overall change of the labeling of metabolite pool is

(31) $$\begin{array}{c} \Delta \bar{M}=\Delta \left(cV\bar{m}\right) \end{array}$$

Taking the derivative of it, we have

(32) $$\begin{array}{c} \frac{d\bar{M}}{dt}=\frac{d\left(cV\bar{m}\right)}{dt}=cV\frac{d\bar{m}}{dt}+\bar{m}\frac{d\left(cV\right)}{dt} \end{array}$$

The metabolite pool size is only affected by the net production flux

(33) $$\begin{array}{c} \Delta \left(cV\right)=\int_{0}^{T}f_{Net\_Pro}Vdt\\ \frac{d\left(cV\right)}{dt}=f_{Net\_Pro}V \end{array}$$

Taking Equation (33) into Equation (32), we have

(34) $$\begin{array}{c} \frac{d\bar{M}}{dt}=cV\frac{d\bar{m}}{dt}+f_{Net\_Pro}\bar{m}V \end{array}$$

Combining Equation (34) and Equation (30), we have

(35) $$\begin{array}{c} \overset{n}{\underset{i=1}{\sum }}\left[f_{i}\left(m_{i}-\bar{m}\right)V\right]=cV\frac{d\bar{m}}{dt} \end{array}$$

Divide both sides with *cV*, we have

(36) $$\begin{array}{c} \frac{d\bar{m}}{dt}=\overset{n}{\underset{i=1}{\sum }}\left[f_{i}\left(m_{i}-\bar{m}\right)\right]/c \end{array}$$

**Figure 5:**
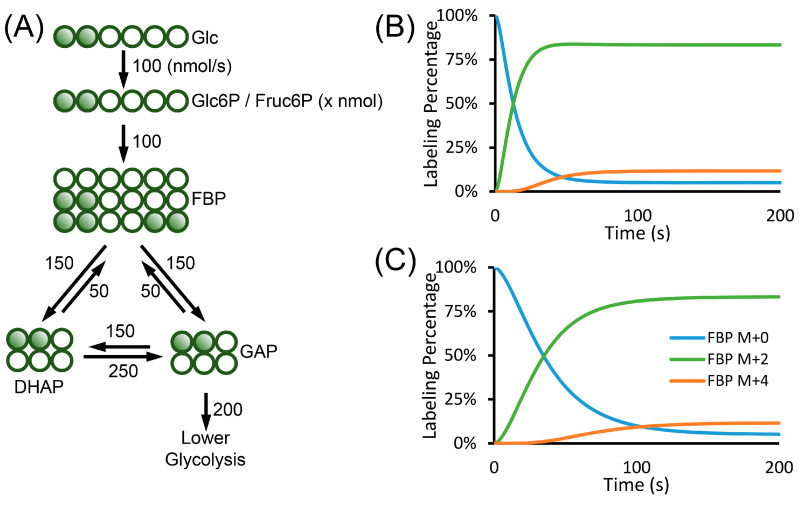
Example of isotopically non-stationary MFA. (**A**) Upper glycolysis network, the carbon sources were switched from non-labeled glucose to 1,2-^13^C_2_-glucose to measure the kinetics of metabolite labeling. The numbers next to the arrows are fluxes in the unit of nmol/s. (**B**,**C**) In isotopically non-stationary MFA, the labeling patterns of metabolite is affected by the pool size of the metabolites. FBP, DHAP and GAP all have the pool size of 1 µmol. The pool size of Glc6P is assumed to be 400 nmols (**B**) or 3000 nmols (**C**). (**B**) FBP in the network with small Glc6P pool size reaches steady state faster. (**C**) FBP in the network with large Glc6P pool size reaches steady state slower. The abbreviations used in the figure are: Glc, glucose; Glc6P, glucose-6-phosphate; Fruc6P, fructose-6-phosphate; FBP, fructose 1,6-bisphosphate; DHAP, dihydroxyacetone phosphate and GAP, glyceraldehyde-3-phosphate.

Equation (36) has the same form as Equation (28). The term *c* in Equation (36) is a function of time, whereas it is a constant in Equation (28). Equation (36) is the general equation describing the flux and the change in the labeling patterns. It applies to biological systems with changing cell numbers and intracellular metabolite concentrations. Similar to the metabolic and isotopic steady state case in Equation (10), the changes in the labeling patterns are largely affected by the input fluxes. The consumption fluxes are not included in the equation. They can only affect the concentration term c to impact the labeling changes. Comparing Equations (10) and (36), we also see that the metabolite concentration under isotopic steady state is not related to the flux and the labeling patterns. However, the concentration term determines how fast the labeling can change under non-steady state condition. The change of pool size in one metabolite will also result in the changes in the labeling kinetics of other metabolites. For example, when the Glc6P pool expands, the FBP labeling also becomes slower. However, when the isotopic steady state is reached, the pool size of Glc6P does not affect the labeling pattern of FBP (Figure 5).

To solve the fluxes under isotopic non-steady state, we also take the simulation-optimization approach. Given a set of fluxes and the metabolite concentrations, we can predict the labeling patterns at different time points of sampling. The model predictions are compared to the experimental measurements. The flux combinations that generate the labeling kinetics that match the measurements the best are the optimal solution for the isotopic non-steady state MFA.

## 8. Conclusions

In conclusion, MFA uses stable isotope labeling to solve metabolic fluxes. MFA commonly adopts two assumptions: the metabolites are well-mixed, and the kinetic isotope effect is negligible. Under isotopic steady state, one metabolite’s labeling pattern represents the flux-weighted average of the substrates. Under non-steady state, the rate of change of the labeling pattern is determined by the substrate and product labeling patterns, the production fluxes of this metabolite and the concentration of the metabolite.

## Figures and Tables

**Table 1:** Commonly used software tools for metabolic flux analysis (MFA).

Name	Data Source	Main Features	Number of Citations	Reference and Most Cited Applications
13CFLUX2	MS and NMR	Compatible to multi-platform	107	[39,40,41,42]
FiatFlux	GC-MS	^13^C Glucose tracer, flux ratio	160	[43,44,45,46]
INCA	MS and NMR	Isotopically non-stationary MFA	147	[47,48,49,50,51]
METRAN	MS	Intuitive graphical user interface, confidence interval calculation	183	[52,53,54]
NMR2Flux+	NMR	NMR data, plant network	124	[55,56,57,58]
OpenFLUX	MS	Steady-state ^13^C MFA, experimental design	154	[59,60,61,62]
OpenMebius	MS	Isotopically non-stationary MFA	47	[63,64,65]

## Code Availability

All the code used to generate graphs can be accessed at https://github.com/XiaoyangSu/MFA-Demo.

## References

[1] Boyle J. (2005). Lehninger Principles of Biochemistry (4th ed.): Nelson, D., and Cox, M. Biochem. Mol. Biol. Educ..

[2] Caspi R., Altman T., Billington R., Dreher K., Foerster H., Fulcher C.A., Holland T.A., Keseler I.M., Kothari A., Kubo A. (2014). The MetaCyc database of metabolic pathways and enzymes and the BioCyc collection of Pathway/Genome Databases. Nucleic Acids Res..

[3] Fiehn O. (2002). The link between genotypes and phenotypes. Plant Mol. Biol..

[4] Beckonert O., Keun H.C., Ebbels T.M.D., Bundy J., Holmes E., Lindon J.C., Nicholson J.K. (2007). Metabolic profiling, metabolomic and metabonomic procedures for NMR spectroscopy of urine, plasma, serum and tissue extracts. Nat. Protoc..

[5] Hollywood K., Brison D.R., Goodacre R. (2006). Metabolomics: Current technologies and future trends. Proteomics.

[6] Nicholson J.K., Lindon J.C. (2008). Systems biology: Metabonomics. Nature.

[7] Holmes E., Antti H. (2002). Chemometric contributions to the evolution of metabonomics: Mathematical solutions to characterising and interpreting complex biological NMR spectra. Analyst.

[8] Hoult D.I., Busby S.J.W., Gadian D.G., Radda G.K., Richards R.E., Seeley P.J. (1974). Observation of tissue metabolites using nuclear magnetic resonance. Nature.

[9] Griffiths W.J., Wang Y. (2009). Mass spectrometry: From proteomics to metabolomics and lipidomics. Chem. Soc. Rev..

[10] Gomez-Casati D.F., Zanor M.I., Busi M.V. (2013). Metabolomics in plants and humans: Applications in the prevention and diagnosis of diseases. Biomed Res. Int..

[11] Swanson M.G., Zektzer A.S., Tabatabai Z.L., Simko J., Jarso S., Keshari K.R., Schmitt L., Carroll P.R., Shinohara K., Vigneron D.B. (2006). Quantitative analysis of prostate metabolites using 1H HR-MAS spectroscopy. Magn. Reson. Med..

[12] Kline E.E., Treat E.G., Averna T.A., Davis M.S., Smith A.Y., Sillerud L.O. (2006). Citrate Concentrations in Human Seminal Fluid and Expressed Prostatic Fluid Determined via 1H Nuclear Magnetic Resonance Spectroscopy Outperform Prostate Specific Antigen in Prostate Cancer Detection. J. Urol..

[13] Sauer U. (2006). Metabolic networks in motion: ^13^C-based flux analysis. Mol. Syst. Biol..

[14] Jang C., Chen L., Rabinowitz J.D. (2018). Metabolomics and Isotope Tracing. Cell.

[15] Jeremy J.Y., Ballard S.A., Naylor A.M., Miller M.A.W., Angelini G.D. (1997). Effects of sildenafil, a type-5 cGMP phosphodiesterase inhibitor, and papaverine on cyclic GMP and cyclic AMP levels in the rabbit corpus cavernosum in vitro. Br. J. Urol..

[16] Wiechert W. (2001). ^13^C metabolic flux analysis. Metab. Eng..

[17] Antoniewicz M.R. (2015). Methods and advances in metabolic flux analysis: A mini-review. J. Ind. Microbiol. Biotechnol..

[18] Dai Z., Locasale J.W. (2017). Understanding metabolism with flux analysis: From theory to application. Metab. Eng..

[19] Zamboni N. (2011). ^13^C metabolic flux analysis in complex systems. Curr. Opin. Biotechnol..

[20] Cascante M., Selivanov V., Ramos-Montoya A. (2012). Application of tracer-based metabolomics and flux analysis in targeted cancer drug design. Methods Pharmacol. Toxicol..

[21] Antoniewicz M.R. (2018). A guide to ^13^C metabolic flux analysis for the cancer biologist. Exp. Mol. Med..

[22] Boghigian B.A., Seth G., Kiss R., Pfeifer B.A. (2010). Metabolic flux analysis and pharmaceutical production. Metab. Eng..

[23] Warburg O., Posener K., Negelein E. (1924). The metabolism of cancer cells. Biochem. Z.

[24] Vander Heiden M.G., Cantley L.C., Thompson C.B. (2009). Understanding the Warburg effect: The metabolic requirements of cell proliferation. Science.

[25] Faubert B., Li K.Y., Cai L., Hensley C.T., Kim J., Zacharias L.G., Yang C., Do Q.N., Doucette S., Burguete D. (2017). Lactate Metabolism in Human Lung Tumors. Cell.

[26] Hui S., Ghergurovich J.M., Morscher R.J., Jang C., Teng X., Lu W., Esparza L.A., Reya T., Zhan L., Yanxiang Guo J. (2017). Glucose feeds the TCA cycle via circulating lactate. Nature.

[27] Van Gulik W.M., Antoniewicz M.R., deLaat W.T.A.M., Vinke J.L., Heijnen J.J. (2001). Energetics of growth and penicillin production in a high-producing strain of Penicillium chrysogenum. Biotechnol. Bioeng..

[28] Orman M.A., Berthiaume F., Androulakis I.P., Ierapetritou M.G. (2011). Advanced stoichiometric analysis of metabolic networks of mammalian systems. Crit. Rev. Biomed. Eng..

[29] Carinhas N., Bernal V., Teixeira A.P., Carrondo M.J., Alves P.M., Oliveira R. (2011). Hybrid metabolic flux analysis: Combining stoichiometric and statistical constraints to model the formation of complex recombinant products. BMC Syst. Biol..

[30] Feist A.M., Palsson B.O. (2010). The biomass objective function. Curr. Opin. Microbiol..

[31] Orth J.D., Thiele I., Palsson B.O. (2010). What is flux balance analysis?. Nat. Biotechnol..

[32] Ranganathan S., Suthers P.F., Maranas C.D. (2010). OptForce: An optimization procedure for identifying all genetic manipulations leading to targeted overproductions. PLoS Comput. Biol..

[33] Raghunathan A., Shin S., Daefler S. (2010). Systems approach to investigating host-pathogen interactions in infections with the biothreat agent Francisella. Constraints-based model of Francisella tularensis. BMC Syst. Biol..

[34] Harcombe W.R., Delaney N.F., Leiby N., Klitgord N., Marx C.J. (2013). The ability of flux balance analysis to predict evolution of central metabolism scales with the initial distance to the optimum. PLoS Comput. Biol..

[35] Bonarius H.P.J., Timmerarends B., De Gooijer C.D., Tramper J. (1998). Metabolite-balancing techniques vs. ^13^C tracer experiments to determine metabolic fluxes in hybridoma cells. Biotechnol. Bioeng..

[36] Schmidt K., Marx A., De Graaf A.A., Wiechert W., Sahm H., Nielsen J., Villadsen J. (1998). ^13^C tracer experiments and metabolite balancing for metabolic flux analysis: Comparing two approaches. Biotechnol. Bioeng..

[37] Çalik P., Akbay A. (2000). Mass flux balance-based model and metabolic flux analysis for collagen synthesis in the fibrogenesis process of human liver. Med. Hypotheses.

[38] Reisz J.A., D’Alessandro A. (2017). Measurement of metabolic fluxes using stable isotope tracers in whole animals and human patients. Curr. Opin. Clin. Nutr. Metab. Care.

[39] Weitzel M., Nöh K., Dalman T., Niedenführ S., Stute B., Wiechert W. (2013). 13CFLUX2—High-performance software suite for ^13^C-metabolic flux analysis. Bioinformatics.

[40] Beste D.J.V., Nöh K., Niedenführ S., Mendum T.A., Hawkins N.D., Ward J.L., Beale M.H., Wiechert W., McFadden J. (2013). ^13^C-flux spectral analysis of host-pathogen metabolism reveals a mixed diet for intracellular mycobacterium tuberculosis. Chem. Biol..

[41] Chen Z., Bommareddy R.R., Frank D., Rappert S., Zeng A.P. (2014). Deregulation of feedback inhibition of phosphoenolpyruvate carboxylase for improved lysine production in Corynebacterium glutamicum. Appl. Environ. Microbiol..

[42] Liu L., Shah S., Fan J., Park J.O., Wellen K.E., Rabinowitz J.D. (2016). Malic enzyme tracers reveal hypoxia-induced switch in adipocyte NADPH pathway usage. Nat. Chem. Biol..

[43] Zamboni N., Fischer E., Sauer U. (2005). FiatFlux—A software for metabolic flux analysis from ^13^C-glucose experiments. BMC Bioinform..

[44] Wang Q., Zhang Y., Yang C., Xiong H., Lin Y., Yao J., Li H., Xie L., Zhao W., Yao Y. (2010). Acetylation of metabolic enzymes coordinates carbon source utilization and metabolic flux. Science.

[45] Del Castillo T., Ramos J.L., Rodríguez-Herva J.J., Fuhrer T., Sauer U., Duque E. (2007). Convergent peripheral pathways catalyze initial glucose catabolism in Pseudomonas putida: Genomic and flux analysis. J. Bacteriol..

[46] Fong S.S., Nanchen A., Palsson B.O., Sauer U. (2006). Latent pathway activation and increased pathway capacity enable Escherichia coli adaptation to loss of key metabolic enzymes. J. Biol. Chem..

[47] Young J.D. (2014). INCA: A computational platform for isotopically non-stationary metabolic flux analysis. Bioinformatics.

[48] Lewis C.A., Parker S.J., Fiske B.P., McCloskey D., Gui D.Y., Green C.R., Vokes N.I., Feist A.M., Vander Heiden M.G., Metallo C.M. (2014). Tracing Compartmentalized NADPH Metabolism in the Cytosol and Mitochondria of Mammalian Cells. Mol. Cell.

[49] Jiang L., Shestov A.A., Swain P., Yang C., Parker S.J., Wang Q.A., Terada L.S., Adams N.D., McCabe M.T., Pietrak B. (2016). Reductive carboxylation supports redox homeostasis during anchorage-independent growth. Nature.

[50] Vacanti N.M., Divakaruni A.S., Green C.R., Parker S.J., Henry R.R., Ciaraldi T.P., Murphy A.N., Metallo C.M. (2014). Regulation of substrate utilization by the mitochondrial pyruvate carrier. Mol. Cell.

[51] Green C.R., Wallace M., Divakaruni A.S., Phillips S.A., Murphy A.N., Ciaraldi T.P., Metallo C.M. (2016). Branched-chain amino acid catabolism fuels adipocyte differentiation and lipogenesis. Nat. Chem. Biol..

[52] Yoo H., Antoniewicz M.R., Stephanopoulos G., Kelleher J.K. (2008). Quantifying reductive carboxylation flux of glutamine to lipid in a brown adipocyte cell line. J. Biol. Chem..

[53] Metallo C.M., Gameiro P.A., Bell E.L., Mattaini K.R., Yang J., Hiller K., Jewell C.M., Johnson Z.R., Irvine D.J., Guarente L. (2012). Reductive glutamine metabolism by IDH1 mediates lipogenesis under hypoxia. Nature.

[54] Noguchi Y., Young J.D., Aleman J.O., Hansen M.E., Kelleher J.K., Stephanopoulos G. (2009). Effect of anaplerotic fluxes and amino acid availability on hepatic lipoapoptosis. J. Biol. Chem..

[55] Sriram G., Fulton D.B., Iyer V.V., Peterson J.M., Zhou R., Westgate M.E., Spalding M.H., Shanks J.V. (2004). Quantification of compartmented metabolic fluxes in developing soybean embryos by employing biosynthetically directed fractional ^13^C labeling, two-dimensional [^13^C, 1H] nuclear magnetic resonance, and comprehensive isotopomer balancing. Plant Physiol..

[56] Murarka A., Clomburg J.M., Moran S., Shanks J.V., Gonzalez R. (2010). Metabolic analysis of wild-type Escherichia coli and a Pyruvate Dehydrogenase Complex (PDHC)-deficient derivative reveals the role of PDHC in the fermentative metabolism of glucose. J. Biol. Chem..

[57] Fu Y., Yoon J.M., Jarboe L., Shanks J.V. (2015). Metabolic flux analysis of Escherichia coli MG1655 under octanoic acid (C8) stress. Appl. Microbiol. Biotechnol..

[58] Iyer V.V., Sriram G., Fulton D.B., Zhou R., Westgate M.E., Shanks J.V. (2008). Metabolic flux maps comparing the effect of temperature on protein and oil biosynthesis in developing soybean cotyledons. Plant Cell Environ..

[59] Quek L.E., Wittmann C., Nielsen L.K., Krömer J.O. (2009). OpenFLUX: Efficient modelling software for ^13^C-based metabolic flux analysis. Microb. Cell Fact..

[60] Nocon J., Steiger M.G., Pfeffer M., Sohn S.B., Kim T.Y., Maurer M., Rußmayer H., Pflügl S., Ask M., Haberhauer-Troyer C. (2014). Model based engineering of Pichia pastoris central metabolism enhances recombinant protein production. Metab. Eng..

[61] Bommareddy R.R., Chen Z., Rappert S., Zeng A.P. (2014). A de novo NADPH generation pathway for improving lysine production of Corynebacterium glutamicum by rational design of the coenzyme specificity of glyceraldehyde 3-phosphate dehydrogenase. Metab. Eng..

[62] Buschke N., Becker J., Schäfer R., Kiefer P., Biedendieck R., Wittmann C. (2013). Systems metabolic engineering of xylose-utilizing Corynebacterium glutamicum for production of 1,5-diaminopentane. Biotechnol. J..

[63] Kajihata S., Furusawa C., Matsuda F., Shimizu H. (2014). OpenMebius: An Open Source Software for Isotopically Nonstationary ^13^C-Based Metabolic Flux Analysis. Biomed Res. Int..

[64] Miyazawa H., Yamaguchi Y., Sugiura Y., Honda K., Kondo K., Matsuda F., Yamamoto T., Suematsu M., Miura M. (2017). Rewiring of embryonic glucose metabolism via suppression of PFK-1 and aldolase during mouse chorioallantoic branching. Development.

[65] Wada K., Toya Y., Banno S., Yoshikawa K., Matsuda F., Shimizu H. (2017). ^13^C-metabolic flux analysis for mevalonate-producing strain of Escherichia coli. J. Biosci. Bioeng..

[66] Antoniewicz M.R., Kelleher J.K., Stephanopoulos G. (2007). Elementary metabolite units (EMU): A novel framework for modeling isotopic distributions. Metab. Eng..

[67] Wiechert W., Möllney M., Isermann N., Wurzel M., De Graaf A.A. (1999). Bidirectional reaction steps in metabolic networks: III. Explicit solution and analysis of isotopomer labeling systems. Biotechnol. Bioeng..

[68] Simmons E.M., Hartwig J.F. (2012). On the interpretation of deuterium kinetic isotope effects in C-H bond functionalizations by transition-metal complexes. Angew. Chemie. Int. Ed..

[69] Liuni P., Olkhov-Mitsel E., Orellana A., Wilson D.J. (2013). Measuring kinetic isotope effects in enzyme reactions using time-resolved electrospray mass spectrometry. Anal. Chem..

[70] Tea I., Tcherkez G. (2017). Natural Isotope Abundance in Metabolites: Techniques and Kinetic Isotope Effect Measurement in Plant, Animal, and Human Tissues. Methods in Enzymology.

[71] Roth J.P., Klinman J.P. (2013). Kinetic Isotope Effects. Encyclopedia of Biological Chemistry.

[72] Westheimer F.H. (1961). The magnitude of the primary kinetic isotope effect for compounds of hydrogen and deuterium. Chem. Rev..

[73] Fan J., Ye J., Kamphorst J.J., Shlomi T., Thompson C.B., Rabinowitz J.D. (2014). Quantitative flux analysis reveals folate-dependent NADPH production. Nature.

[74] Williams T.C.R., Sweetlove L.J., George Ratcliffe R. (2011). Capturing metabolite channeling in metabolic flux phenotypes. Plant Physiol..

[75] Spivey H.O., Ovádi J. (1999). Substrate channeling. Methods A Companion Methods Enzymol..

[76] Miles E.W., Rhee S., Davies D.R. (1999). The molecular basis of substrate channeling. J. Biol. Chem..

[77] Zhang Y.H.P. (2011). Substrate channeling and enzyme complexes for biotechnological applications. Biotechnol. Adv..

[78] Wahrheit J., Nicolae A., Heinzle E. (2011). Eukaryotic metabolism: Measuring compartment fluxes. Biotechnol. J..

[79] Allen D.K., Shachar-Hill Y., Ohlrogge J.B. (2007). Compartment-specific labeling information in ^13^C metabolic flux analysis of plants. Phytochemistry.

[80] Toledano M.B., Delaunay-Moisan A., Outten C.E., Igbaria A. (2013). Functions and cellular compartmentation of the thioredoxin and glutathione pathways in yeast. Antioxid. Redox Signal..

[81] Antoniewicz M.R., Kelleher J.K., Stephanopoulos G. (2006). Determination of confidence intervals of metabolic fluxes estimated from stable isotope measurements. Metab. Eng..

[82] Metallo C.M., Walther J.L., Stephanopoulos G. (2009). Evaluation of ^13^C isotopic tracers for metabolic flux analysis in mammalian cells. J. Biotechnol..

[83] Crown S.B., Long C.P., Antoniewicz M.R. (2016). Optimal tracers for parallel labeling experiments and ^13^C metabolic flux analysis: A new precision and synergy scoring system. Metab. Eng..

[84] Nargund S., Sriram G. (2013). Designer labels for plant metabolism: Statistical design of isotope labeling experiments for improved quantification of flux in complex plant metabolic networks. Mol. Biosyst..

[85] Crown S.B., Ahn W.S., Antoniewicz M.R. (2012). Rational design of ^13^C-labeling experiments for metabolic flux analysis in mammalian cells. BMC Syst. Biol..

[86] Millard P., Sokol S., Letisse F., Portais J.C. (2014). IsoDesign: A software for optimizing the design of ^13^C-metabolic flux analysis experiments. Biotechnol. Bioeng..

[87] Isermann N., Wiechert W. (2003). Metabolic isotopomer labeling systems. Part II: Structural flux identifiability analysis. Math. Biosci..

[88] Kadirkamanathan V., Yang J., Billings S.A., Wright P.C. (2006). Markov Chain Monte Carlo Algorithm based metabolic flux distribution analysis on Corynebacterium glutamicum. Bioinformatics.

[89] Yang J., Wongsa S., Kadirkamanathan V., Billings S.A., Wright P.C. (2005). Metabolic flux distribution analysis by^13^C-tracer experiments using the Markov chain-Monte Carlo method. Biochem. Soc. Trans..

[90] Buescher J.M., Antoniewicz M.R., Boros L.G., Burgess S.C., Brunengraber H., Clish C.B., DeBerardinis R.J., Feron O., Frezza C., Ghesquiere B. (2015). A roadmap for interpreting ^13^C metabolite labeling patterns from cells. Curr. Opin. Biotechnol..

[91] Ma F., Jazmin L.J., Young J.D., Allen D.K. (2014). Isotopically nonstationary ^13^C flux analysis of changes in Arabidopsis thaliana leaf metabolism due to high light acclimation. Proc. Natl. Acad. Sci. USA.

[92] Yuan J., Bennett B.D., Rabinowitz J.D. (2008). Kinetic flux profiling for quantitation of cellular metabolic fluxes. Nat. Protoc..

[93] Noack S., Nöh K., Moch M., Oldiges M., Wiechert W. (2011). Stationary versus non-stationary ^13^C-MFA: A comparison using a consistent dataset. J. Biotechnol..

[94] Nöh K., Wiechert W. (2006). Experimental design principles for isotopically instationary ^13^C labeling experiments. Biotechnol. Bioeng..

[95] Nöh K., Grönke K., Luo B., Takors R., Oldiges M., Wiechert W. (2007). Metabolic flux analysis at ultra short time scale: Isotopically non-stationary ^13^C labeling experiments. J. Biotechnol..

